# Pseudoaneurysm of the Cystic Artery: A Unique Presentation of a Ruptured Gallbladder

**DOI:** 10.7759/cureus.94751

**Published:** 2025-10-16

**Authors:** Hamdan Mallick, Jerome Lee, Mridul Pansari, Thomas Barnett

**Affiliations:** 1 Medicine, Bayhealth Hospital, Karachi, PAK; 2 Surgery, Bayhealth Hospital, Dover, USA

**Keywords:** cystic artery pseudoaneurysm, lap chole, perforated gall bladder, pseudoaneurysm, rupture

## Abstract

This case report details the atypical presentation and management of a 65-year-old male with a medical history encompassing hypertension, hepatitis C, and alcohol abuse (Model for End-Stage Liver Disease (MELD) score: 6). The patient presented to the emergency department with chest and lower abdominal pain, nausea, and vomiting. Initial imaging, including a contrast-enhanced computed tomography angiogram (CTA), revealed a distended gallbladder, perihepatic fluid, and an intraluminal blush arising from the right cystic artery. Laparoscopic cholecystectomy was performed, revealing a substantial perihepatic blood collection and a ruptured gallbladder with a posteriorly positioned cystic artery that was seen entering the gallbladder lumen through the posterior wall. Bleeding was observed filling into the gallbladder. Owing to significant adhesions and gallbladder friability, a subtotal cholecystectomy was undertaken, with clips applied and electrocautery employed for hemostasis. The cystic artery was clipped at its takeoff and subsequently cauterized. Postoperatively, minimal serosanguineous drainage was observed, and pathological examination of the gallbladder confirmed a benign nature. The case discussion addresses the rarity of gallbladder perforation, especially in the context of acute cholecystitis, and introduces a unique scenario of an abnormally positioned cystic artery causing a pseudoaneurysm, a novel phenomenon not previously documented. The critical presentation precluded cystic artery embolization, necessitating a tailored approach with a subtotal cholecystectomy. This case contributes valuable insights into the understanding and management of complex gallbladder pathologies, emphasizing the importance of adapting surgical strategies to address multifaceted anatomical and clinical considerations.

## Introduction

This case report examines a rare presentation of gallbladder pathology in a 65-year-old male with a history of hypertension, hepatitis C, and alcohol abuse. Pseudoaneurysms of the cystic artery are uncommon vascular lesions, most often arising in the setting of acute cholecystitis, trauma, or cholecystectomy. The incidence of cystic artery pseudoaneurysms is estimated to be less than 1% of all cases of acute cholecystitis. These pseudoaneurysms typically present with right upper quadrant pain, jaundice, and hemobilia, though symptoms can be nonspecific, making diagnosis challenging. The presence of a cystic artery pseudoaneurysm can lead to life-threatening hemorrhage, requiring urgent surgical intervention.

Gallbladder perforation is a well-recognized but rare complication of acute cholecystitis. It is most commonly associated with cholelithiasis and can result in biliary peritonitis, septic shock, and multiorgan failure. The cystic artery typically runs in Calot's triangle, superomedially to the cystic duct. The presence of a posteriorly positioned cystic artery is a less common anatomical variation. This report highlights a case of a ruptured gallbladder with an intraluminal cystic artery pseudoaneurysm, a presentation not previously described in the literature.

## Case presentation

**Figure 1:**
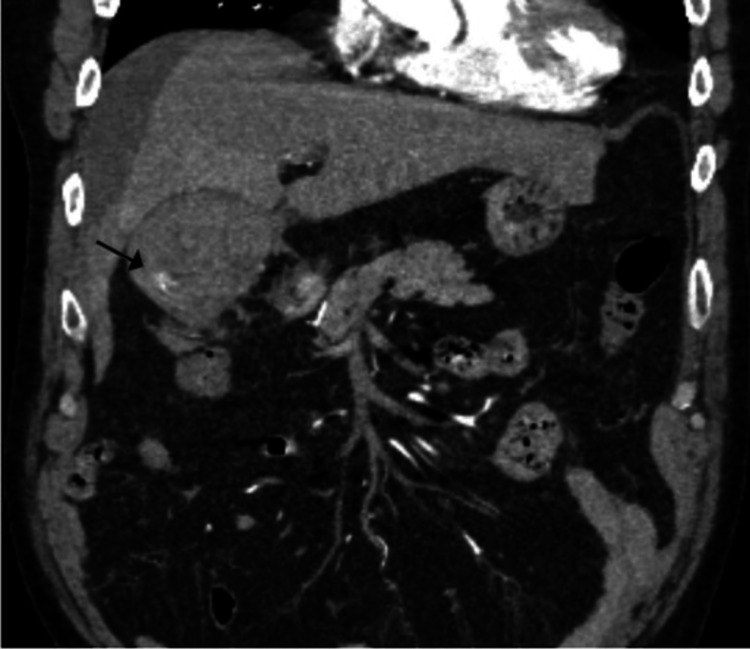
Cystic artery pseudoaneurysm (black arrow)

**Figure 2:**
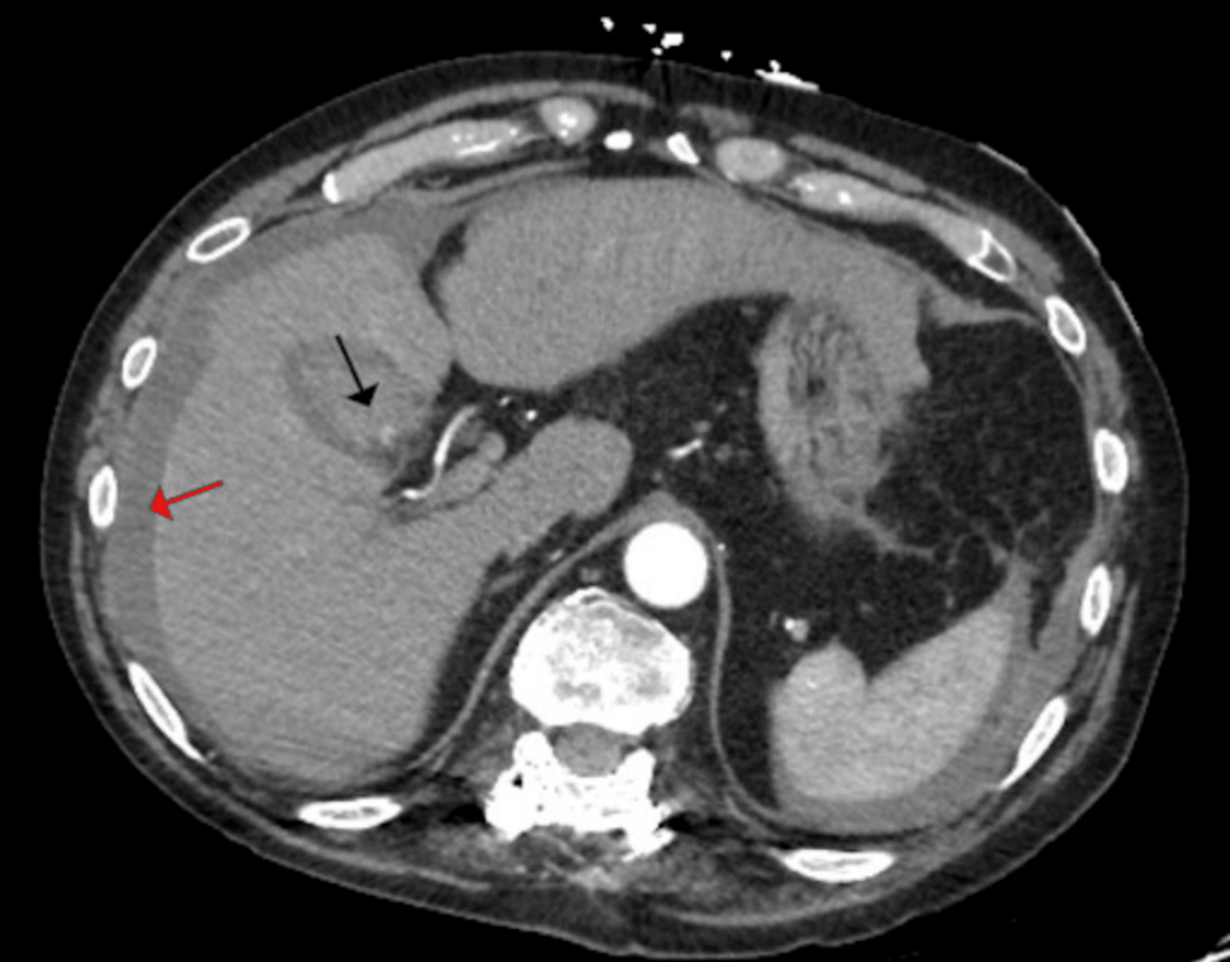
Dense perihepatic fluid consistent with blood (red arrow). Pseudoaneurysm within gallbladder is seen as a blush (black arrow)

A 65-year-old male with a history of hypertension, hepatitis C, and an alcohol abuse score (Model for End-Stage Liver Disease (MELD)) of 6 presented to the emergency department with chest and lower abdominal pain associated with nausea and vomiting. During the initial workup, a computed tomography angiogram (CTA) was performed to rule out pulmonary embolism. The scan revealed a distended gallbladder, significant perihepatic fluid, and stones. Notably, abnormal contrast enhancement was observed arising from the cystic artery, appearing as an intraluminal blush in the gallbladder (Figures 1, 2). 

The density of blood observed in the gallbladder lumen and perihepatic regions was 50 HU, raising suspicion for possible blood contents. An ultrasound was subsequently performed, which confirmed the presence of gallstones and debris filling the gallbladder lumen. The collection density was similar to that of the hemoperitoneum seen on CT. Importantly, the patient denied any recent trauma or use of blood thinners. Laboratory workup results are shown in Table 1.

**Table 1:** Patient laboratory results WBC: white blood cell, AST: aspartate aminotransferase, INR: international normalized ratio.

Laboratory Study	Laboratory Result	Reference Range
WBC	11,300 cells/µL	4,500-11,000 cells/µL
Hemoglobin	12.3 g/dL	13.5-17.5 g/dL
AST	30 U/L	8-48 U/L
Total bilirubin	0.8 mg/dL	0.1-1.2 mg/dL
INR	1.1	0.8-1.2
Lipase	82 U/L	0-160 U/L

**Figure 3:**
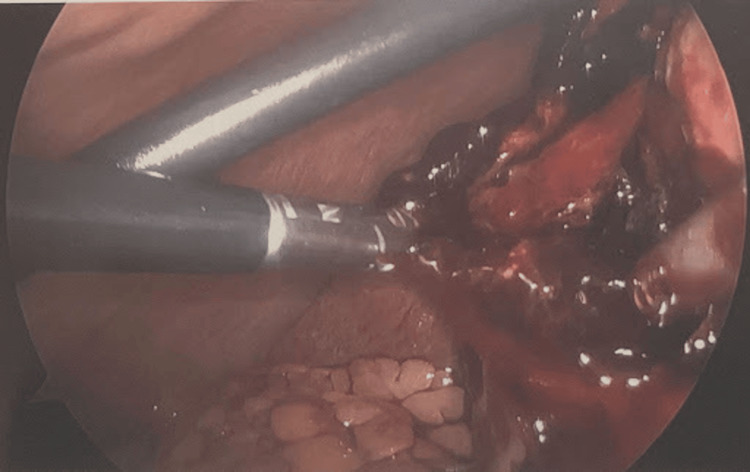
Ruptured gallbladder with a large rent

The patient continued to have episodic tachycardia at 100-120 beats per minute, with systolic pressure in the 90s, despite adequate resuscitation. Given the patient's imaging findings, vital signs, and positive Murphy’s sign, a decision was made to proceed with laparoscopic cholecystectomy. Upon entry into the abdomen, a significant collection of perihepatic blood was visualized and suctioned out, totaling 700 cc (Figure 3).

A collapsed gallbladder was observed containing a large rent in the fundus with fresh blood oozing (Figure 3). Given the significant adhesions and friable nature of the gallbladder, a subtotal cholecystectomy was performed. The gallbladder was very friable, and clips were applied along with electrocautery for hemostasis. Upon reaching the critical angle, the cystic artery was seen entering posteriorly into the back wall of the gallbladder, with continued oozing of blood from this area. The cystic artery was then identified at its takeoff and subsequently cauterized. The position of the cystic duct takeoff could not be safely visualized; therefore, the operation was concluded with a subtotal approach. Hemostasis was achieved, and a drain was left in place. On postoperative follow-up, the JP drain output after one week was noted to be minimal and serosanguineous. Surgical pathology of the gallbladder revealed findings consistent with cholecystitis.

## Discussion

Perforation of the gallbladder is a rare presentation in the setting of acute cholecystitis. In our unique case, we observed a type II Niemeier perforation that was likely secondary to acute cholecystitis accompanied by intraluminal gallbladder bleeding. Gallbladder perforation is almost always associated with cholelithiasis and carries a significant mortality risk of 10% [1]. Our case was notable in that intraluminal bleeding may also have played a role.

Although cholecystectomy is a relatively standardized procedure, abnormal anatomical variations of the cystic artery are common. It is described as the second most common anatomical variation of the hepatic pedicle. A large data-based study by Andall et al. revealed that only 81.5% of cystic arteries were found in the cystic triangle. They were typically observed to run anterior or to the right of the common hepatic duct [2]. This was not the case in our patient, where the cystic artery was found traversing posteriorly and entering laterally into the gallbladder.

Cystic artery embolization was not possible as a bridging measure, given that the patient presented with acute cholecystitis and hemodynamic instability. Cystic artery pseudoaneurysms themselves are uncommon occurrences, typically developing after acute cholecystitis or cholecystectomy. The classical triad, known as Quincke’s triad, includes jaundice, right upper quadrant pain, and upper gastrointestinal bleeding [3,4]. In a literature review by Fujimoto et al., the majority of patients presented with right upper quadrant pain and hemobilia as the most common findings. Most of these cases were treated with cystic artery embolization, which was not feasible in our patient due to the critical nature of his presentation [3]. With regard to perforated gallbladders, they are typically suspected based on sonographic findings of non-shadowing, non-mobile intraluminal echogenic material [4]. Suspicion was confirmed in our case with CT, which revealed high attenuation within the gallbladder and a blush within its lumen [5].

A review by Taghavi et al. found that over 60% of cystic artery pseudoaneurysms were associated with acute cholecystitis, suggesting a potential inflammatory etiology rather than a coincidental anatomical anomaly. This supports the possibility that localized inflammation may have contributed to the formation of a pseudoaneurysm in our patient [6].

No case has been reported in the literature describing a posteriorly positioned cystic artery with a pseudoaneurysm perforating into the gallbladder, resulting in rupture. This was a highly complex case due to multiple coexisting anatomical and clinical rarities. Addressing the gallbladder aggressively with a subtotal cholecystectomy and hemostatic control proved to be a successful approach.

## Conclusions

This case report highlights the atypical presentation and management of a ruptured gallbladder with a cystic artery pseudoaneurysm, a novel phenomenon not previously documented. Despite the challenges posed by significant adhesions and gallbladder friability, a tailored approach using subtotal cholecystectomy was successfully employed, emphasizing the importance of adapting surgical strategies to address multifaceted anatomical and clinical considerations in complex gallbladder pathologies.
